# Chromatin and transcriptome changes in human myoblasts show spatio-temporal correlations and demonstrate DPP4 inhibition in differentiated myotubes

**DOI:** 10.1038/s41598-020-70756-x

**Published:** 2020-08-31

**Authors:** Tomasz J. Kolanowski, Natalia Rozwadowska, Agnieszka Zimna, Magdalena Nowaczyk, Marcin Siatkowski, Wojciech Łabędź, Ewa Wiland, Jacek Gapiński, Stefan Jurga, Maciej Kurpisz

**Affiliations:** 1 Institute of Human Genetics Polish Academy of Sciences, Poznan, Poland; 2 Institute of Biostatistics and Informatics in Medicine and Ageing Research, Medical Faculty, University of Rostock, Rostock, Germany; 3 Department of Spondyloorthopaedics and Biomechanics of Spine, W. Dega University Hospital, University of Medical Sciences, Poznan, Poland; 4 NanoBioMedical Center, University of Adam Mickiewicz, Poznan, Poland

**Keywords:** Nuclear organization, Cytogenetics, Muscle stem cells

## Abstract

Although less attention was paid to understanding physical localization changes in cell nuclei recently, depicting chromatin interaction maps is a topic of high interest. Here, we focused on defining extensive physical changes in chromatin organization in the process of skeletal myoblast differentiation. Based on RNA profiling data and 3D imaging of myogenic (*NCAM1, DES, MYOG, ACTN3, MYF5, MYF6, ACTN2,* and *MYH2*) and other selected genes (*HPRT1, CDH15, DPP4* and *VCAM1*), we observed correlations between the following: (1) expression change and localization, (2) a gene and its genomic neighbourhood expression and (3) intra-chromosome and microscopical locus-centromere distances. In particular, we demonstrated the negative regulation of *DPP4* mRNA (p < 0.001) and protein (p < 0.05) in differentiated myotubes, which coincided with a localization change of the *DPP4* locus towards the nuclear lamina (p < 0.001) and chromosome 2 centromere (p < 0.001). Furthermore, we discuss the possible role of DPP4 in myoblasts (supported by an inhibition assay). We also provide positive regulation examples (*VCAM1* and *MYH2*). Overall, we describe for the first time existing mechanisms of spatial gene expression regulation in myoblasts that might explain the issue of heterogenic responses observed during muscle regenerative therapies.

## Introduction

The changes in the localization of chromosomes and their genes in the three-dimensional nuclear space are tightly regulated but highly variable phenomena. These nuclear interactions have an impact on transcriptional regulation, cell fate and inheritance mechanisms^1–3^.

Recent years have led to the development of many high-throughput techniques that have vastly increased our knowledge and understanding of chromatin interaction mechanisms^4–7^. However, these high-throughput techniques (called ‘3C techniques’) rely on sequence proximity in order to map ongoing interactions. However, creating a physical map of chromatin positioning in relation to the other components of nuclei remains a challenge. To define specific position changes of chromatin in nuclei, microscopic observations of labelled material are necessary, and these techniques have been developed simultaneously with 3C techniques, ultimately combining both approaches^8–12^.

Human muscle-derived stem cells have been used in regenerative therapies for nearly two decades. Since pluripotent stem cell (iPSC)-based technology is still lacking clinical feasibility, myoblast-based therapies can be considered generally safe and effective for regaining muscle contraction after sphincter rupture or when used in experimental therapies of muscle dystrophies^13^. One of the difficulties of cellular therapies is patient variability and response, which diminishes the general therapeutic effect. The pro-regenerative abilities of the myoblast (Mb) population are believed to be age related and can be affected by injuries and muscle-related diseases^14,15^.

Moreover, these cells are interesting because of their specific differentiation process. In damaged or exercised striated muscle-activated satellite cells, myoblasts (Mb) become mitotically active. After a number of divisions, they start the process of fusion, creating multinuclear myotubes (Mt). This terminal differentiation leads to the generation of a specific contraction machinery involved in forming myofibers, with nuclei localized peripherally near the cell membrane^16^. A cascade of transcription factors among which are the Pax, MRF (myogenic regulatory factors) and Mef-2 families drives the described process. Pax7^+^ satellite cells remain quiescent at birth. Upon activation, transcription factors from the MRF family are expressed in apical cells after asymmetric division. Next, Pax7^+^/Mef5^+^ satellite cells undergo symmetric division^17^. Second, an increase in the MyoD level leads to further proliferation and subsequent activation of myogenin (MyoG). MyoG, together with Mef-2c (from the Mef-2 family), can be observed after cell cycle exit, myoblast fusion and creation of multinucleated myotubes^18^. The maturation of the contraction apparatus is driven by Myf6 and MyoG and is associated with a diminished level of the other transcription factors^19^. Despite the clinical use of skeletal myoblasts and the changes during their differentiation, the process has not gained much attention thus far in terms of nuclear changes, which might underlie the changes in cell performance with age.

Dipeptidyl peptidase IV (DPPIV, CD26) is a membrane protein involved in glucose metabolism that inactivates incretins with its peptidase activity^20^. Incretins are proteins released by the gastrointestinal system in response to digestion processes. Incretins induce increased levels of circulating insulin and decrease glucagon release, which leads to increased glucose uptake by the liver and other tissues (ex. skeletal muscle) thus diminishing glucose blood levels. In particular, two major incretins, intestinal glucagon-like peptide-1 (GLP-1) and gastric inhibitory peptide (GIP), are both hydrolysed by DPP4. This robust reaction led to the development of DPP4 inhibitors, which are drugs used for the treatment of diabetes type 2^21^. Apart from its role in glucose metabolism, DPP4 is a signal transducing protein that can modulate proline/alanine-containing peptides, including growth factors and vasoactive peptides^22,23^.

In our previous work, we described alterations in centromere position in human skeletal myoblasts during their differentiation process together with changes in the general expression pattern^24^. Here, we focused on the characterization of gene-specific localization changes in the nuclear landscape and the correlation with their expression levels. Therefore, we could confirm a tendency of transcriptionally activated loci for preferential localization in the middle zone between the nucleus border and its centre, and that transcriptional inactivation led to increased contact with the nuclear lamina. Moreover, we observed and measured the relationship between the changes in centromere-gene loci distance and gene expression, which did not confirm an earlier stated mechanism. Additionally, upon in-depth analysis of nuclear localization and other levels of regulation, we observed that DPP4 is downregulated in differentiating skeletal myoblasts. Based on previously published data and our own experiments, we hypothesized that DPP4 might serve as a factor in the negative feedback loop controlling the effect of insulin on the proliferation of myoblasts.

## Results

### Characterization of the human myoblast population

**Figure 1:**
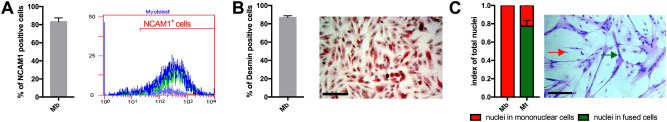
Evaluation of isolated myoblast populations. (**A**) Flow cytometric analysis of CD56 (NCAM1) expression. The histogram with blue violet and green overlapping lines represent three different samples. (**B**) Immunohistochemistry of desmin (red) expression in a myoblast population; **C** Fusion index by Giemsa (blue) staining shows that approximately 80% of nuclei are fused; multinuclear cells are visible in all isolated populations. Bars indicate 50 µm.

Because human primary skeletal muscle-derived stem cells show high individual variability, we performed all experiments using cells derived from 3 different tissue preparations. Analysis of isolated myoblasts by flow cytometry showed that 86.0 ± 4.2% of cells were NCAM-1 (CD56) positive, which demonstrated high purity of the evaluated samples (Fig. 1A). We also tested the desmin (DES) content, showing 86.9 ± 2% desmin-positive cells (Fig. 1B) in the analysed sample. Moreover, the fusion index value (0.77 ± 0.06) revealed that almost 80% of nuclei were located in fused cells after differentiation (Fig. 1C). These characterization steps demonstrated that the investigated populations had high purity, high myoblast marker gene expression and robustly mimicked physiological processes.

### Nuclear morphology

The nuclei morphology measurements of each nucleus were done being a crucial normalization parameter for all subsequent distance measurements and to confirm our previous results. To evaluate nuclear morphology of differentiated myotubes (Mt) and actively dividing myoblasts (Mb), we measured their size (volume) and shape (flattening), as we have previously described^24^. Here, we confirmed that cells in the differentiated population (Mt) were smaller 811.1 ± 231.1 μm^3^ and more elongated (− 0.935 ± 0.308; less spheroidal) when compared to their undifferentiated counterparts (970.9 ± 341.3 μm^3^ and − 0.771 ± 0.286, respectively). In both cases, we found statistical significance at p < 0.001 with a total n > 600 for the analysed parameters and groups (Supplementary Figure S1).

### Gene expression analysis and rationale for target selection

**Figure 2:**
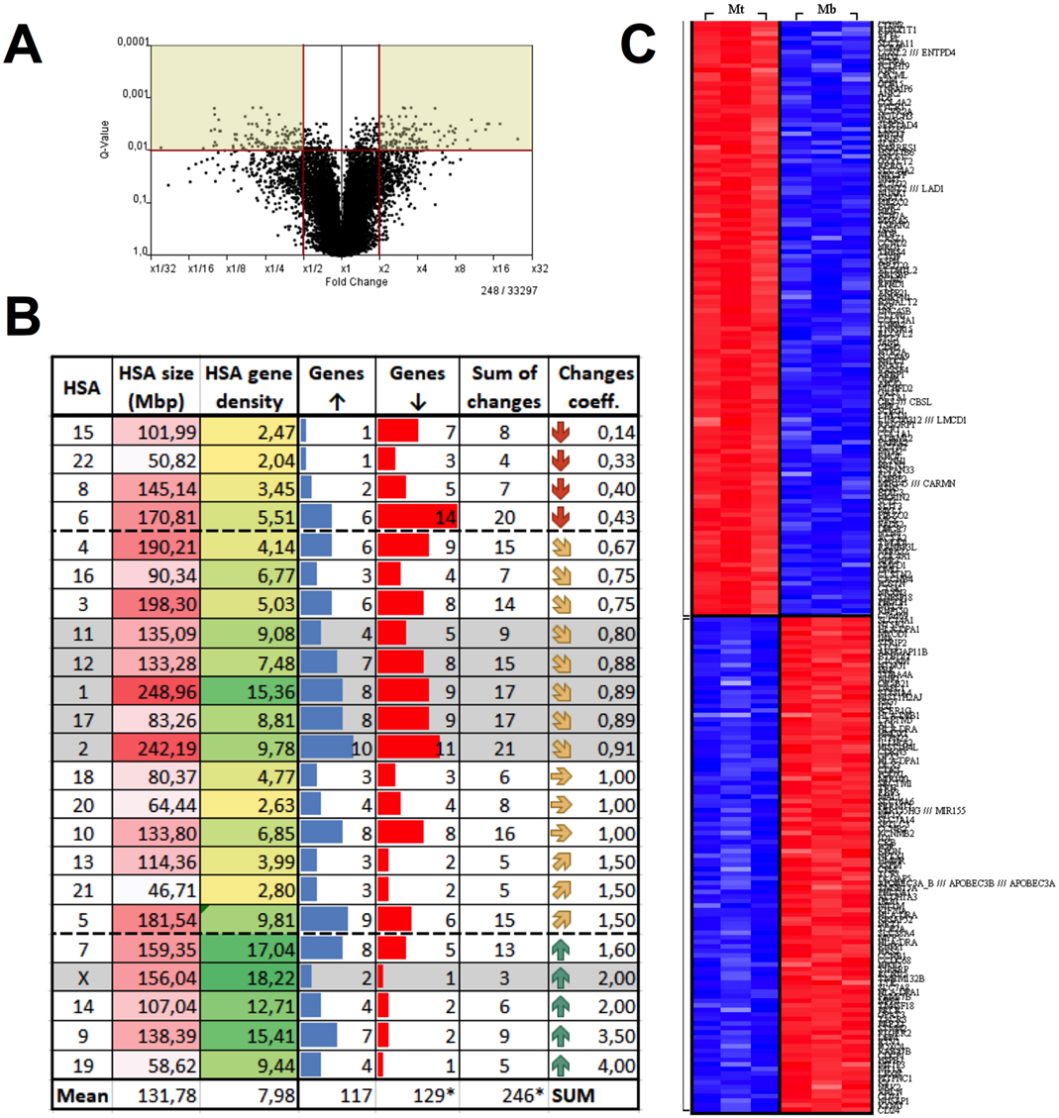
Statistical evaluation of microarray data. (**A**) volcano plot showing exclusion criteria. (**B**) Chromosomes with annotated genes that undergo statistically significant changes during the differentiation process. In total, 246 genes with significant changes were detected. Chromosomes were grouped according to the change coefficient into three groups: mainly downregulated (red arrow), mainly upregulated (green arrow) and a third group with intermediate, mixed pattern (yellow arrow). For further analysis, genes from the most active chromosomes from the intermediate group were selected (marked by grey background). As a control, chromosome X with the HPRT gene was selected. (C) Heat map representation of all differentially expressed genes in myoblasts (Mb) and myotubes (Mt).

Comparison of expression profiles by microarray technique showed that 246 transcripts were differentially expressed (p < 0.01), with 117 transcripts showing at least twofold higher expression in Mt and 129 transcripts with expression at least twofold lower (downregulated) than in Mb (Fig. 2A,C). Next, we divided the differentially expressed transcripts (both up- and downregulated) by their genomic localization in chromosomes (Fig. 2B) and grouped chromosomes by the values of the change coefficient (0.5 < C_co_ < 1.5). Selecting chromosomes with intermediate C_co_ values allowed us to avoid extreme situations (such as chromosomes that might be prone to inactivation, which would result in biased specific sequence positions). Among the chromosomes with intermediate C_co_, we selected the most active chromosomes with the highest number of differences that were shown to include both up- and downregulated genes. Within this group, we found large (e.g., 1) and small (e.g., 17) HAS, as well as chromosomes that had gene density above (e.g., 1) and below (e.g., 12) the average for the human chromosome set. This approach allowed us to select active chromosomes and avoid those that simply did not show differences in the myotube formation process.

**Figure 3:**
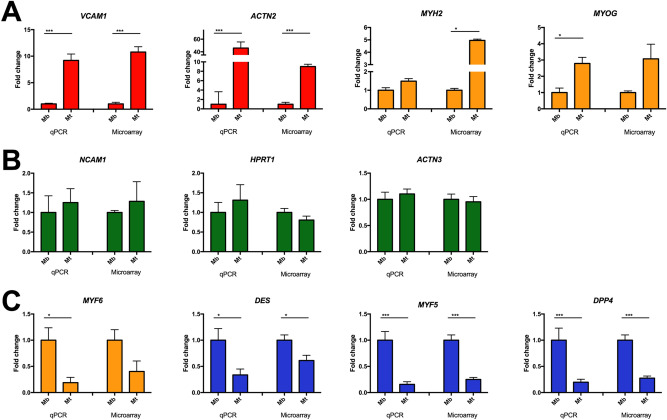
Confirmation of expression changes obtained by microarray and qPCR analyses. (**A**) Genes upregulated in myotubes (Mt) showed statistical significance by both methods (red) or only one method (orange). (**B**) Genes that remained stable during differentiation. (**C**) Genes downregulated in Mt confirmed by both methods (blue) or only one method (orange). Asterisks: *p < 0.05; **p < 0.01; ***p < 0.001.

Interestingly, within the selected chromosome group, we found several myogenic genes that showed different expression patterns during the process of myoblast differentiation (Fig. 3). These included transcription factors known to drive the myoblast differentiation process: *MYF6, MYF5* and *MYOG*. Additionally, we found structural sarcomeric genes (*MYH2, ACTN2 and ACTN3* were selected) and known markers of myoblasts (*NCAM1* and *DES*). Moreover, among the genes with the most pronounced expression changes, we decided to evaluate the genomic position of two genes—upregulated (*VCAM1*) and downregulated (*DPP4*). Although these genes showed different expression patterns, all of them were in the analysed group of highly active chromosomes (Fig. 3A–C).

### Clusters of expression

**Figure 4:**
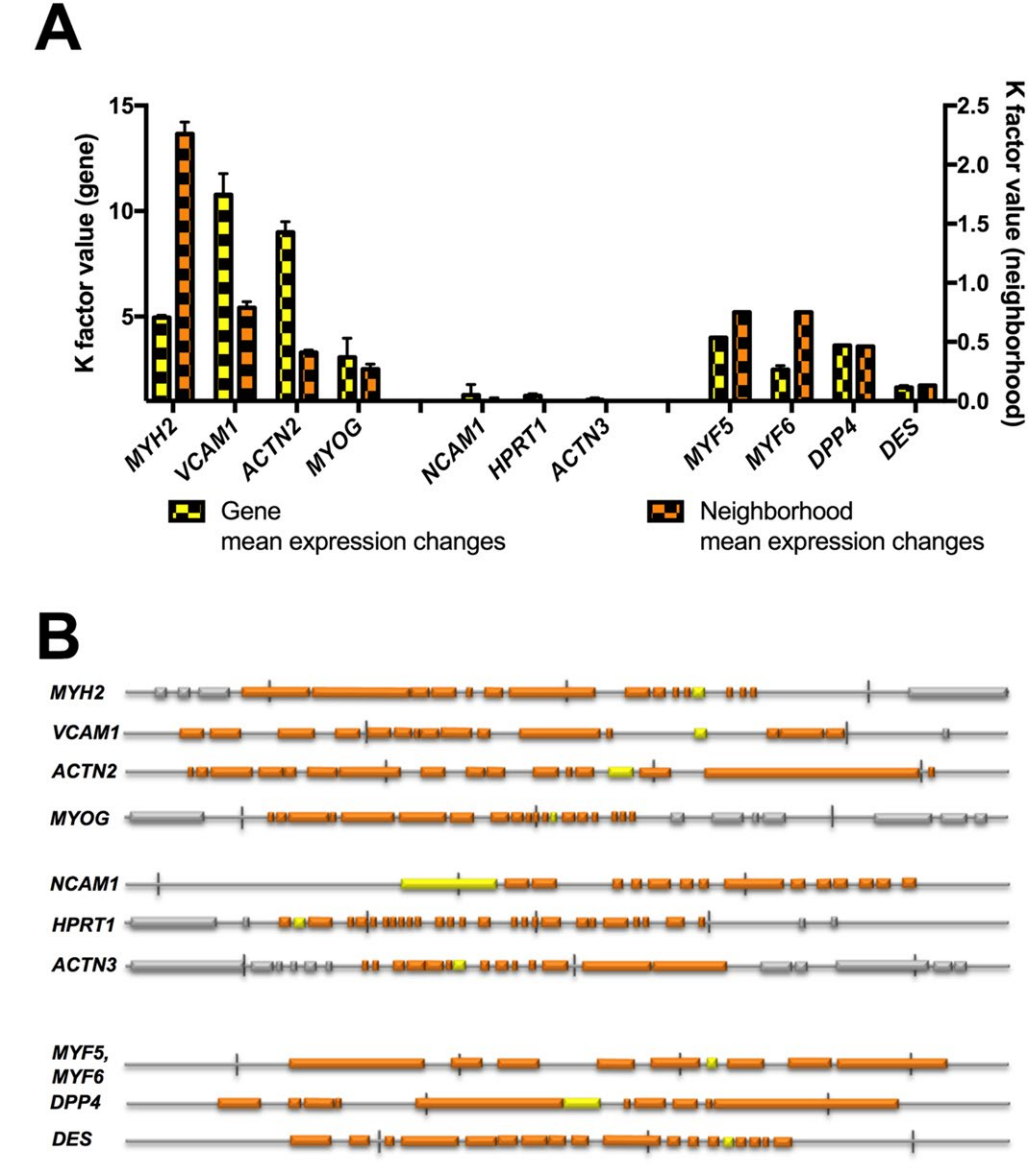
Analysis of proximal chromatin expression activity of selected genes. (**A**) Mean expression changes in genes correlated with increased activity in the gene neighbourhood. In contrast, genes that did not show expression changes (*NCAM1*, *HPRT1*, *ACTN3*) resided in a stable neighbourhood. (**B**) Selected neighbourhood of approximately 1 Mbp size for each selected gene.

To show the relative expression changes in neighbouring genomic regions of the selected genes, we used K factor values (defined in the Materials & Methods section). We observed that expression changes in the selected genes (K_GOI_) were correlated with differences within their neighbourhood as defined by the mean K value (K_mean;_ Spearman correlation r = 0.83, 95% CI: 0.45–0.96; p = 0.0025). Both up- and downregulated genes appeared to reside in the regions that were actually prone to changes (*MYH2, VCAM1, ACTN2, MYOG, MYH5, MYH6, DPP4* and *DES*). On the other hand, most of the genes with stable expression during the differentiation process were retained in a stable neighbourhood (*NCAM1, HPRT1* and *ACTN3*; Fig. 4).

### Changes in physical positions of *loci* of interest within cell nuclei

**Figure 5:**
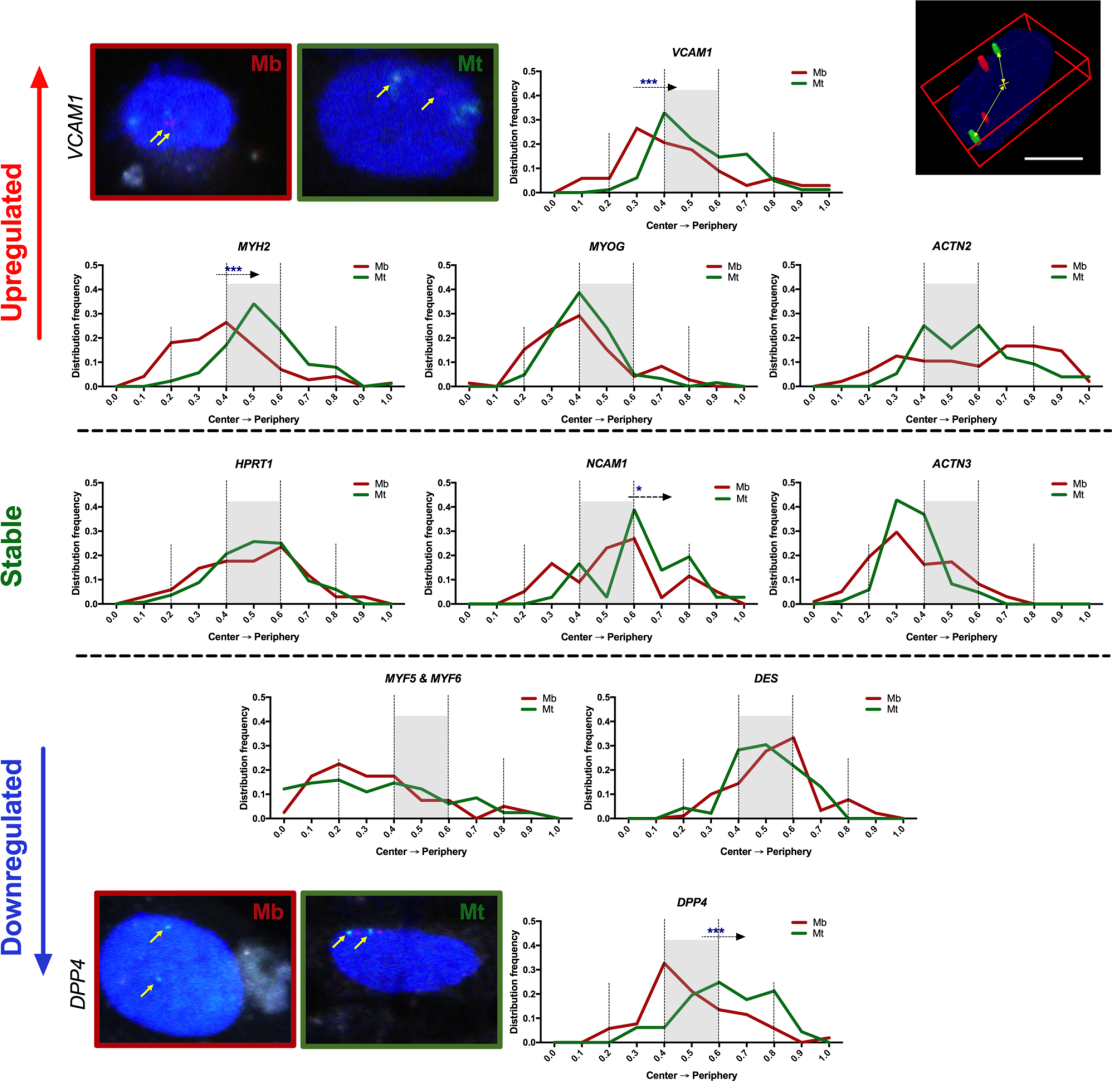
Localization changes of selected GOIs before and after differentiation of human myoblasts. GOI probe localization is shown in terms of distribution from the nuclear centre (0.0) to the nuclear periphery (1.0), also called normalized distance index. Loci with increased expression (red) show a general tendency to move towards the middle zones between the nuclear centre and periphery. Loci of genes that did not undergo expression changes (green) resided mainly in the same area. Downregulated genes (blue) had the tendency to reside or move towards extreme zones: periphery (like *DPP4*) or nuclear centre (in the case of *MYH5* and *MYH6*). Asterisks: *p < 0.05; ***p < 0.001; arrows show the direction of the observed localization change.

Measurements of the position of gene-specific probes in nuclei showed significant changes in 4 out of 10 cases. These were *MYH2, VCAM1, DPP4* (p < 0.001) and *NCAM1* (p < 0.01) (Fig. 5 & Supplementary Figure S2). For genes that were upregulated during differentiation, *MYH2* and *VCAM1* localization seemed to change to a more intermediate position (central between the nuclear centre and periphery). *MYOG* showed a similar pattern of localization changes as *VCAM1;* however, we did not observe statistical significance in this case. *ACTN* showed a different migration pattern—from the nuclear periphery back to the middle zones; however, no statistical significance was observed.

Interestingly, in the case of *DPP4,* one of the most pronounced downregulated genes, we observed behaviour opposite to that of *ACTN*. *DPP4* was dislocated from the middle zones in myoblasts outwards to the peripheral parts of the nucleus in myotubes (Fig. 5 & Supplementary Figure S2). The intensity of *DPP4* dislocation towards the nuclear lamina in the MT group was confirmed by immunoFISH staining (Supplementary Figure S3). Two other downregulated genes resided in close intra-chromosomal proximity to each other (analysed by one probe covering both sequences), and they were retained in the centric zone of the nuclei. Desmin loci were observed in the middle zone with little change towards the centre of the nuclei.

*HPRT1*, a control gene with stable expression during the differentiation process, remained in the middle zone with almost no differences in localization distribution. *ACTN3* did not significantly alter its localization; however, it was closer to the middle zones in Mt. The only GOI with unchanged expression but altered distribution was *NCAM1* (Figs. 3, 5, Supplementary Table S1).

These results suggest the existence of highly active transcription sites in the intermediate positions (middle zones) and places of diminished expression at the nuclear periphery, which was visible in differentiated myotubes (Mt).

### Changes in heterochromatin localization during cell differentiation

Because selected chromosomes were shown to be highly active according to their expression changes, we tested whether these changes could also influence their internal constitutive heterochromatin regions. To do so, we picked the most stable heterochromatin regions—centromeres—and evaluated their position changes. Of the 6 observed centromeres, three showed significant dislocation. These were centromeres of large metacentric chromosomes 1 (p < 0.05), 2 (p < 0.01) and of acrocentric chromosome 17 (p < 0.001; Supplementary Figure S4. In all of these cases, centromeres relocated from the nuclear centre towards the periphery, preferentially locating in the middle zones. Interestingly, regardless of their size, these chromosomes contain more genes with altered expression during differentiation (HAS 1, 2, and 17 have > 17 changes) when compared to the remaining 3 (11, 12 and X have < 15 changes) from selected group of six. Centromeres of chromosomes with a lower number of expression changes within the selected group—11 and X did not change their position, remaining in the middle zone between the nuclear centre and periphery. The centromeric regions of HAS12 remained closer to the nuclear centre (Supplementary Figure S4, Supplementary Table S2). This might suggest that due to the intensive changes of the chromosomal euchromatin, also its heterochromatin might undergo relocation.

### Other position changes and interactions within cell nuclei

**Figure 6:**
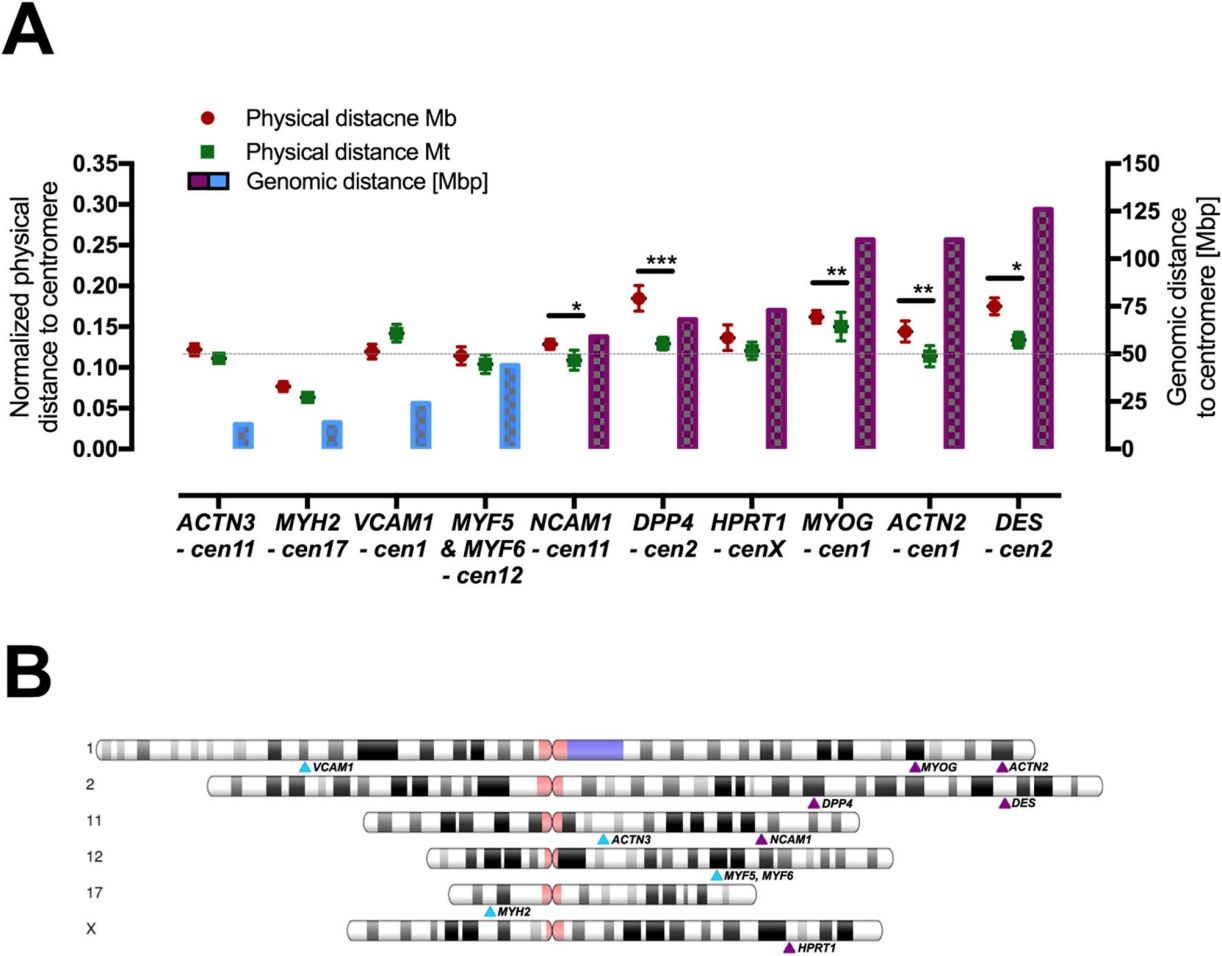
Comparison of normalized distance index between GOIs and chromosome centromere changes during cell differentiation to intra-chromosome distance (localization in chromosome counted by mega base pairs, Mbp) from the chromosome centromere centre. (**A**) Statistically significant changes in distances due to myoblast differentiation were significant for loci whose intra-chromosome distance from the centromere exceeded 50 Mbp. For lower intra-chromosome distances, no significant changes were observed. (**B**) Chromosome map with the analysed loci marked. Loci with intra-chromosome distances larger than 50 Mbp are marked with violet arrows; others are marked with light blue arrows. Asterisks: *p < 0.05; **p < 0.01; ***p < 0.001.

To elucidate the parameter that might influence the localization changes in the analysed sequences, we compared the distance index and intra-chromosomal distances (measured in Mbp) of selected genes to their centromere signals. In fact, almost all genes (apart from *HPRT*) with longer intra-chromosomal distances showed significant changes; however, when the intra-chromosomal distance decreased to < 50 Mbp, the distance index changes were no longer observed (Fig. 6). Although none of the selected genes were strictly associated with heterochromatin of the centromere at any point of the analysis, it would be important to further elucidate whether these changes in distances could have some effect on gene expression.

### *DPP4* expression

**Figure 7:**
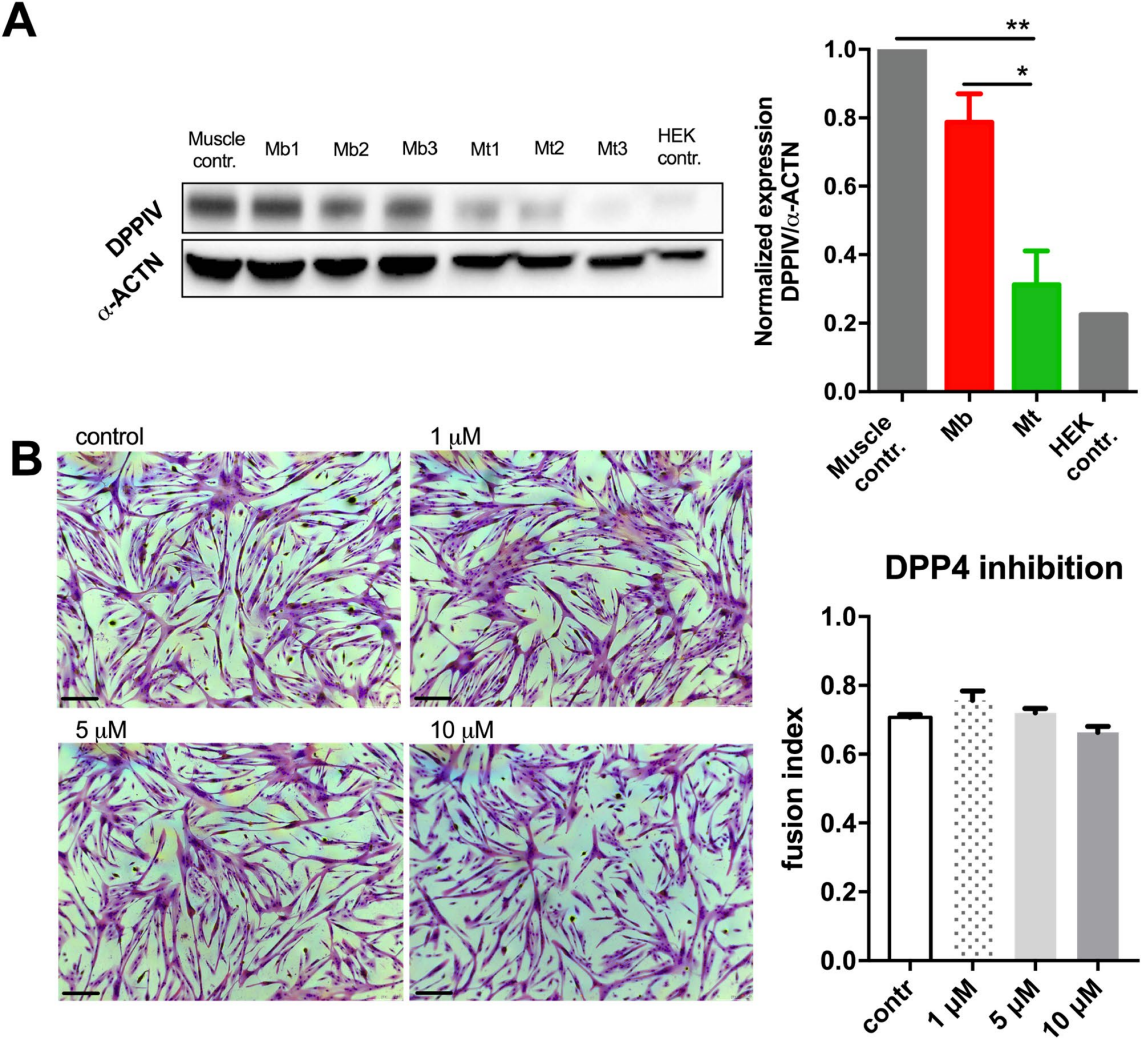
Evaluation of DPP4 presence and function during the differentiation process of myoblasts. (**A**) Western blot showing decreased expression of DPP4 after myotube formation (chart on the right). All original full-length blots are presented in Supplementary Figure S6. (**B**) Evaluation of differentiation efficiency with treatment with DPP4 inhibitor (sitagliptin) by measuring the fusion index (chart on the right). Cells were treated with 1, 5 or 10 µM f sitagliptin and simultaneously subjected to a differentiation protocol. Images show fused, multinucleated myotubes. Sitagliptin used at 1 µM conc. seemed to increase fusion; however, no significant differences were observed (see images and chart). Asterisks: *p < 0.05; **p < 0.01; bars indicate 50 µm.

Most of the genes evaluated in this study are known to directly regulate or to be involved in the course of the differentiation process. *DPP4,* however, is one of the most negatively regulated genes in myotubes, as confirmed by high-throughput expression and qPCR experiments (both methods indicated approximately fivefold downregulation, Fig. 3). Moreover, the *DPP4* locus revealed a vast change in its nuclear position during differentiation towards the nuclear periphery with close proximity to the nuclear lamina, as shown by the immunoFISH technique (Supplementary Figure S3). Taking into consideration the transmembrane and/or extracellular position of the protein product of the *DPP4* gene and thus its potential as a marker gene, we decided to further study this phenomenon. We confirmed DPP4 downregulation at the protein level in myotubes (Fig. 7A & Supplementary Figure S7). To test the effect of chemically induced DPP4 inhibition of myoblast differentiation, we supplemented the myocyte differentiation medium with sitagliptin. Initially, we performed toxicity studies, which showed that concentrations above 50 µM were not suitable for analyses due to increased cell death. Nevertheless, in the range of 1 to 10 µM, the DPP4 inhibitor resulted in a rather insignificant increase (treatment with 0.1 µM Sit) in the fusion index of myotube cultures (Fig. 7B & Supplementary Figure S7).

## Discussion

In this study, we examined the nuclear localization changes of selected loci and their correlation with expression pattern changes during the process of human skeletal muscle differentiation. We showed that genes with high expression reside in the middle zone between the nuclear centre and lamina and that control of gene expression might be related to a physical change in the localization of the loci. We also tested several hypotheses concerning nuclear architecture. Moreover, we found and described the mechanism of *DPP4* downregulation in differentiated myotubes. DPP4 is a membrane protein expressed in myoblasts, which we propose as a promising new marker of myoblast differentiation among the other markers used to retrospectively describe the initial cell population. Deep characterization of the differentiation process is crucial to overcome known therapeutic difficulties of myoblast therapy caused by population variability. Our results support the resolution of these difficulties in terms of cell growth and regenerative capacity.

First, as primary myoblast populations have been isolated by the preplate technique, we characterized the obtained population by the well-known marker of the myoblast population NCAM1 (over 85% of cells were NCAM1^+^) and the cytoskeletal protein desmin (approx. 87%) proving the purity of the studied samples^25–27^. Moreover, the efficiency of myoblast-specific physiological ability for fusion into myotubes was evaluated as well. The value of the fusion index (0.77) is consistent with previous reports showing that approximately 80% of satellite cells in the muscle readily enter the differentiation process to ultimately fuse and form myotubes. However, up to 20% of the cells remain as a reserve population due to the presence of symmetric and asymmetric division^28^. As known from Cheng et al., the kinetics of myoblast differentiation reach a plateau between days 6 and 8, which additionally confirms that cells differentiated by our protocol remained in the late phases of differentiation^29^.

Apart from fusion and general morphological changes, we confirmed in an earlier study that during myoblast specialization, myoblast nuclei decrease and become more elongated (Supplementary Figure S1)^24^. According to one of the theories, myotubes gain highly organized sarcomeric and cytoskeletal structures that fill almost the entire cell, which leads to lateralization of nuclear position and enforces shape changes. This theory, however, focuses only on physical changes of the cell structure, not taking into account nuclear stiffness and biological processes in the nucleus. In fact, transcription and regulation of expression as well as cell-specific genetic material organization require certain physical space to function. This observation is more prominent when comparing the nuclear sizes of pluripotent cells with more differentiated cells, such as fibroblasts, whereby the latter have smaller, more compact nuclei^30^. In this particular case, earlier findings suggested that the heterochromatin amount in myotubes is increased after differentiation^31^. Increased amounts of compacted chromatin can explain the decreased need for space and thus different nuclear sizes. It would be interesting to compare the nonspecific neighbourhood of DNA strands (by ‘C’ techniques) between two populations of Mb and Mt to determine if these changes lead to a significant increase in chromatin packaging concentration.

The initial selection of the chromosomes in this study was based on the general number of statistically significant changes observed by mRNA analysis. This was meant to avoid any predominantly regulated regions that could bias our observations. In the second stage, genes were selected based on their biological significance for the differentiation process. These selection steps allowed us to focus on the most intriguing loci during differentiation that remained in the regions of high expression plasticity. In fact, we observed changes in nuclear localization in almost all genes that were demonstrated to have altered expression in Mb and Mt. Indeed, many reports have already reported tremendous localization changes of gene position during differentiation of pluripotent stem cells or haematopoietic stem cells^32,33^. Our study, however, is the first to demonstrate this type of regulation in skeletal myoblast terminal differentiation, where the changes are more tenuous and occur in already committed cells. Thus, we provide additional evidence that large spatial gene or chromosome localization changes are much more common and might occur in more subtle transition states than previously reported.

In one of our analyses, we showed that the genomic neighbourhood of the analysed GOIs underwent more intensive expression changes than observed in the stably expressed regions (such as *NCAM1*, *HPRT1* or *ACTN3*). This does not mean that stable genes are not expressed in these cells. In contrast, these genes remain transcriptionally active (such as *NCAM1*); however, their expression does not seem to influence the myoblast differentiation process, and this is true for other genes in close genomic proximity (in the active or silenced state). On the other hand, changes in expression in the neighbourhood of genes with high expression variability, such as *VCAM1* or *MYH2*, are shared by their proximal chromatin region as well (Fig. 4). This observation is in line with the reported features of topologically associated domains (TADs). TADs are approximately 1 Mbp long clusters of genomic sequences separated by boundary regions that were detected using high-throughput genomic techniques^34,35^. The sequences within these clusters are regulated by the same promotor-gene interaction; thus, their regulation pattern is often connected^36–38^. Although we did not evaluate these regions as TADs, they seemed to have similar size and efficiently mimicked the expected functional outcome of TADs. Therefore, it would be highly interesting to follow these observations with chromosome conformation capture methods and define the TAD pattern in myotubes.

Another finding concerns the gene loci-chromosome centromere relationship. It is known that centromeric sequences consist of constitutive chromatin, and many downregulated genes are thought to approximate these sequences^39^. To evaluate this hypothesis, we compared the distance index and intra-chromosomal distances between the centromere and all analysed loci. We observed statistically significant differences in distances only in genes that were positioned over 50 Mbp from the centromere in terms of the intra-chromosomal distance. For genes in closer sequence proximity, we did not observe any significant changes in the distance index from their centromere. Thus, potentially lesser mobility of the chromatin might suggest the existence of a value below which physical elasticity of chromatin decreases. Therefore, any major alteration of the localization of sequences located closer than 50 Mbp would need to be connected with a larger alteration of the whole chromosome territory. This could be an extremely important consequence of nuclear material organization and requires a deeper analysis. Our observations might be useful for further discussion concerning the polymer physics of chromatin, which are undergoing simultaneous exploration of the biological meaning of chromatin organization^40^.

Myogenic stem cells derived from skeletal muscle are frequently used for regenerative therapies. Unfortunately, the high patient-to-patient variability of the therapeutic potential of Mb populations hampers their use as a more common treatment strategy in the case of, for example sphincter ruptures^41^. Therefore, it is important to further investigate the mechanisms of cell differentiation to elucidate these differences. During our analysis, we found a strong downregulation of *DPP4* at the RNA level during myoblast differentiation. Following localization changes in the nuclear landscape, we found that the *DPP4* sequence showed a strong pattern of spatial silencing: moving towards close proximity to the nuclear membrane and direct contact with the nuclear lamina (Fig. 5 and Supplementary Figure S3).

Our results contradict the report of Raschke et al., who showed an increase in DPP4 during myoblast differentiation^42^. These discrepancies might be a consequence of different methodological approaches for the experiments. First, we showed that the analysed populations fully meet the criteria of a high-purity myoblast population (in terms of fusion efficiency, CD56 and desmin expression). In our experience, evaluation of each primary cell isolate is critical for myoblast function since the initial cell population may vary between patients. Additionally, it has been shown that the muscle region of origin could have a tremendous impact on myoblast properties as well. However, in the report of Raschke et al., no information about tissue origin was included. Next, we noted a discrepancy between the definition of myotubes. In our study, myotubes are defined as multinuclear cells of increased size and muscle fibre-like morphology with no proliferative activity. In fact, myotubes are the end-stage form of the myoblast differentiation process. The authors of the cited report did not comment on fusion efficiency of the cells that they used or that any other differentiation quality control was applied, which further complicates our comparison. This further raises concern that the myotubes used in this report might actually partially constitute activated and committed myoblasts, which could be the reason for the constant increase in DPP4 expression over the course of differentiation. The results showed in our report strongly suggest that myoblasts are the major source of DPP4 in skeletal muscle cell culture and that differentiation into myotubes causes a significant drop in *DPP4* expression.

Myoblasts play a regulatory role in muscle tissue turnover and its response to exercise^17^. The exact mechanism of satellite cell activation and proliferation control is unknown. DPP4 is known to play a role in antagonizing insulin signalling by degrading incretins, which stimulate insulin secretion^43^. As insulin provides anabolic signals for striated muscles, which promote myoblast proliferation, DPP4 secretion might function in the negative signal loop after myoblasts become activated to block potential overstimulation of the tissue^44^. Indeed, DPP4 was shown to impair insulin signalling in smooth muscle cells, and its levels were increased in obese subjects causing insulin resistance^45,46^. Moreover, DPP4 inhibitors used in diabetes 2 treatment of elderly patients were demonstrated to prevent sarcopenia^47,48^. In this case, insulin insensitivity causes muscle loss. Inhibition of the insulin antagonist pathway by blocking DPP4 increases insulin signalling levels and cell exposure to insulin. As reported before, insulin might promote myoblast activation and division, which would constitute the sarcopenia preventive effect of DPP4 inhibitors. The described mechanism would further explain the increase in DPP4 observed in myoblast cultures by Raschke et al.^42^ Here, activated myoblasts show a much higher increase in secreted DPP4 than in total protein as a reaction to overstimulation in not fully differentiated culture. To prove this concept, we performed DPP4 inhibitor experiments in our cultures. Indeed, the DPP4 inhibitor did not hamper myotube formation, even slightly increasing the fusion index (not significant, Fig. 7B). This excludes any direct effect of DPP4 during skeletal muscle differentiation, instead suggesting the need for external signalling. A comprehensive study of in vivo DPP4 inhibition is needed. Follow-up experiments should investigate insulin stimulation of muscle satellite cells with genetic lineage tracing to show their proliferation and differentiation output in order to define their contribution to muscle fibres and through that a DPP4 inhibitory effect on muscle turnover, thus proving the published in vitro data^44^. Our results could be further explored by using the CRISPR-GO technique that was recently published by Wang et al.^49^ CRISPR-GO provides the possibility of inducible repositioning of the targeted genomic region in living cells, thus enabling induced silencing of a particular sequence (for example, transitions towards nuclear lamina). This would allow us to confirm our results in *DPP4* and other gene silencing and to define whether *DPP4* should be treated as an effector only or is a more important player in the human myoblast differentiation process.

In summary, we have performed a comprehensive analysis of nuclear organization changes of major genes that control human primary skeletal muscle-derived myoblast differentiation. We have shown that spatial changes occur for the loci of genes that regulate the differentiation process and are most likely the reason for their expression changes. We have reported that localization changes take place in a systematic manner and are common in the process of myoblast differentiation. Data derived by microscopic methods can be used for further bioinformatic analysis of chromosome conformation capture methods to create a physical map of chromatin dynamics in the nucleus. Moreover, based on our analysis, we have shown that *DPP4* gene downregulation is caused at the basic level of chromatin spatial translocation, and this may have further consequences for gene expression. Finally, we propose a new hypothesis for DPP4 function in myoblasts as an effector molecule of a negative feedback loop for insulin-mediated muscle stimulation.

## Materials and methods

The human tissue collection procedure was approved by the Local Bioethical Committee at the Poznan Medical University, and informed consent was obtained from each study participant. In addition, all experiments performed with human materials were in accordance with the relevant guidelines and regulations.

### Human myoblasts—derivation, in vitro culture and characterization

Human myoblast cells were isolated based on a preplating protocol and cultured as previously described^27^. Isolated cells were subjected to approx. three weeks of in vitro culture and at least 3 passages. After culture up-scaling to at least 3 × 10^6^ cells, each population was characterized using flow cytometry (CD56 staining), evaluation of desmin expression and karyotyping. Subsequently, the cells were prepared for differentiation, RNA analysis and fluorescence in situ hybridization. For each investigated cell population, the myoblast fusion index was calculated^27^.

The differentiation protocol of confluent myoblasts in DMEM (Life Technologies, Carlsbad, USA) with 2% horse serum (LONZA, Basel, Switzerland) lasted for 7 days. The medium was changed every third day.

For confirmation of myogenic origin, CD56 (Beckman Coulter, Brea, USA) staining was performed according to the manufacturer’s protocol using flow cytometry. Additionally, immunohistochemistry staining for desmin and lamin A + C was performed. Briefly, cells were washed with PBS, fixed for 15 min at 4 °C in 4% PFA and permeabilized with 0.1% Triton X-100 (Sigma-Aldrich, St. Louis, USA) and then blocked for 30 min in 10% FBS in PBS. Primary antibodies (mouse anti-desmin 1:200, DE-U-10, Sigma-Aldrich, St. Louis, USA; mouse anti-lamin A + C 1:50, JOL2, Abcam, Cambridge, UK) were incubated at 4 °C, o/n. The next day, slides were washed 3 × 5 min in PBS and incubated with the secondary antibodies (goat anti-mouse with TxRed, 1:700, ab6787, Abcam, Cambridge, UK) for 1 h without light.

A fusion index (FI) was also calculated. Briefly, nuclear staining of differentiated myotubes based on 30 min staining in 10% Giemsa solution (Merck Millipore, Darmstadt, Germany) was performed according to the manufacturer’s instructions. FI was calculated as the ratio of the number of nuclei in differentiated myotubes (multinucleated cells) to the number of all counted nuclei.

For binding specificity evaluation of the in-house prepared fluorescent probe, we fixed human control blood mononuclear cells to validate the results with metaphase chromosomes (see Supplementary data). Approximately 40 min before fixation, 10 μg/ml KaryoMAX (Life Technologies, Carlsbad, USA) was added to the cell culture. Fixation was performed starting with cell lysis at 37 °C with 0.4% KCl added dropwise and 20 min of incubation. After centrifugation (450 g, 10 min, 20 °C), cells were fixed using cold MtOH:CH_3_COOH fixative (3:1) added dropwise using vortex and incubated for 20 min. This step was repeated three times, and the cells were seeded on prewashed microscopic slides. To define probe specificity, analysis of at least 20 metaphases were investigated by experienced scientists.

### Preparation of the probes

DNA sequences were obtained from the Human Bacterial Artificial Chromosome Library RP11 (Life Technologies, Carlsbad, USA). After standard bacterial culture, BAC sequences were isolated using a PhasePrep BAC DNA Kit (Sigma Aldrich, St. Louis, USA). For labelling of the DNA sequences, we used random primers and a DNA BioPrime (Life Technologies, Carlsbad, USA) kit. The information on all probes used is listed in Supplementary Table S3. The centromeric and some of the locus-specific probes were supplied from Cytocell (Cambridge, UK) or EmpireGenomis (Buffalo, USA). In-house-prepared probes (see Supplementary Table S1) were evaluated for specificity in standard lymphocyte metaphases (Supplementary Figure S5).

### Fluorescence In situ hybridization

Before analysis, cells were harvested from tissue culture dishes and seeded onto Geltrex-coated (Life Technologies, Carlsbad, USA) coverslips. For fluorescence in situ hybridization experiments, cells were prepared as previously described^24^. For this analysis, we used both commercially available and self-prepared fluorescent probes. The general protocol was similar to the one provided by the manufacturer, and it started with a 2 × SSC wash of fixed cells for 4 min at room temperature (RT). Next, cells were incubated with 10 μg/ml RNase A (Sigma Aldrich, St. Louis, USA) in 2 × SSC for 1 h at 37 °C and washed in 2 × SSC for 4 min at RT. Subsequently, a dehydration procedure was performed using 70%, 85% and 96% ethanol washes for 4 min at RT each. After air-drying, a total of 10 μl hybridization mix (3 μl of each probe + supplied hybridization buffer) was applied, and the cells were covered with a microscopic slide and sealed with FixoGum (Marabu, Tamm, Germany). To equal the temperatures of the probes and slides, the samples were incubated for 10 min at 37 °C, after which a denaturation step was performed. Due to the presence of our own probes, the denaturation protocol was optimized and validated for temperature and timing (Supplementary Figure S6). Hybridization was performed overnight at 37 °C in a humidified hybridization chamber. The next day, after coverslip removal, the samples were washed in 0.4 × SSC, 2 min, 74 °C and subsequently in 0.1% Tween 20 (Sigma Aldrich, St. Louis, USA) in 2 × SSC, 1 min, RT. The last step was the addition of 20 μl of DAPI solution and incubation for 10 min at 4 °C. Samples were observed under 63 × or 100 × objective magnification.

### ImmunoFISH

For additional visualization of lamin A + C, an immunostaining procedure was performed before standard FISH experiments. Cells were fixed on microscopic slides as for the FISH procedure. After washing 3 times in PBS, cell membranes were permeabilized using 0.1% Triton X-100 (Sigma Aldrich, St. Louis, USA) for 15 min. Subsequently, slides were washed and the epitopes were blocked with 10% FBS in PBS, 30 min, RT. Incubation with primary antibody was performed o/n at 4 °C, and after washing with PBS, the secondary antibody was added for 1 h, at RT, in the dark. At the end, the cells were washed with 2 × SSC, and a FISH experiment was subsequently performed.

### Analysis of 3D FISH

We used Axio.Observer1, with Plan Apochromat 63x, NA 1.40 Oil DIC objective and ZEN 2010 software (ZEISS, Jena, Germany). The Z-steps were 0.2 µm (approx. 1.5 × calculated Nyquist sampling density) and pinhole between 1.5–2.5 of Airy units. For each measurement group, 80 ± 29 nuclei were measured. A summary of the acquisition parameters is shown in Table 1.

**Table 1:** Summary of the image acquisition parameters used for 3D confocal imaging.

Channel	Excitation (nm)	Emission filters (nm)	Laser; Laser power (%)	Gain	Pixel size; pixel dwell
FL1	405	410–485	Argon; 2%	939	0.13 µm/0.64 µs
FL2	488	497–559	Argon; 1.8%	893	0.13 µm/0.64 µs
FL3	561	563–747	DPSS 561–10; 3%	799	0.13 µm/0.64 µs

Three-dimensional analysis of 3D FISH stacks was performed using Nemo software (Iannuccelli et al., 2010), which allowed the definition of 3D position vectors of each signal and volume, and automatically calculated the desired distances in a 3-dimensional space. All raw measurements and volume data (distances in µm and volumes in µm^3^) were collected in the MySQL database (Oracle, Redwood City, USA). Statistical analysis was conducted using R software (R Core Team, 2013). The graphical analysis was performed using GraphPad Prism statistical software (Prism Software, Irvine, USA). For analysis requirements, we measured or calculated distances between all signals as elucidated in Supplementary Figure S8. To evaluate probe signal position and for other relative measurements, the results were normalized using nuclear size. Normalization was performed by computationally dividing nuclear volume into 10 000 exclusive, isovolumetric and unicentric spheroids, each centered in the core of the nucleus. The measured signal was then computationally distributed to the sphere it belongs to, based on the ratio of its nucleus centre distance to nucleus radius length that passed through the signal centre. This normalization allowed us to define the signal relative distance to the centre and nuclear periphery and describe it as a normalized distance index (with values from 0.0—nuclear centre; to 1.0—nuclear border). Using index values, we were able to show the signal distribution between the nuclear centre and its border on the 2D chart (Fig. 5—x-axis). The distances between probes (genes or centromeres) were also normalized by the nuclear size and are shown in Fig. 6A (left y-axis).

Flattening was calculated using a known distance, nuclear volume and cross-sectional diameter values. Measurements were conducted according to a previously described protocol^24^, and additional normalization strategies are shown in the supplementary data (Supplementary Figure S8).

The intra-chromosomal distances were collected using NCBI Genome Data Viewer (build: GRCh38.13, https://www.ncbi.nlm.nih.gov/genome/gdv/browser). The distance between position of centromere and gene signals was calculated based on the pre-defined pixel/voxel size for particular settings in the confocal microscope. This distance was subsequently normalized to the Feret distance specific for particular nucleus. Such normalization allow us to avoid influence of general nuclei size changes (within and between the groups) in myoblasts and myotubes that might influence non-specific changes of the distances between sequences. The U-Mann–Whitney test was used to evaluate nuclear observations, and the t-test was applied for other analyses. Unless otherwise stated, all numbers are presented as the mean ± SD.

### Analysis of microarrays

For our analysis, we used our previously published data from the GEO database (GSE45819). The CEL files were analysed with Expression Console Software (Affymetrix, Santa Clara, USA) by using Robust Multi-chip Analysis (RMA) to correct the background. A quantile normalization was performed, and the obtained results were normalized by adjusting a set of data expressed on a logarithmic scale. Subsequent analysis was performed using Subio Software (Subio, Japan). Differential expression analysis was conducted for transcripts showing at least a twofold difference between groups, and statistical significance was determined by the Benjamini–Hochberg algorithm for multiple testing to adjust the P-value defined by the t-test (p < 0.01). For each chromosome, we calculated the change coefficient (C_co_), which was defined as the number of downregulated transcripts divided by the number of upregulated transcripts. The chromosome sizes and gene densities were calculated using data from the Ensembl (ensemble.org) database.

To evaluate the effect of differential expression on the expression of sequence clusters, we equalized the effect of each expression change (up- and downregulation). A K factor was defined as follows:

$$\left\{\begin{array}{ll}K={log}_{2}\left({f}_{c}\right), & \quad for\;{f}_{c}>1;\\ K= {log}_{2}\left(\frac{1}{{f}_{c}}\right), & \quad for\;{f}_{c}\le 1;\end{array}\right.$$

where *f*_*c*_—expression fold change.

The K factor helped us define the tendency of the neighbourhood of each selected gene of interest (GOI) to show an increased level of expression changes. The K factor for all genes in the neighbourhood of each selected GOI was summarized and averaged to show the mean expression change of the neighbourhood area (0.95 ± 0.15 Mbp for MYH2, VCAM1, MYOG, NCAM1, ACTN3, DPP4 and DES and 1.5 ± 0.1 Mbp for ACTN2, HPRT, MYF5 and MYF6)—K_mean_.

### Western Blot procedure

For the Western blot experiments, the cell pellets from skeletal muscles, myoblasts, myotubes, and HEK293T cells (ATCC, Manassas, USA) were dissolved in 8 M urea, 50 mM Tris–HCl, pH 8.0 with 1% SDS (1:1) containing protease inhibitor cocktail (Roche, Basel, Switzerland). The total protein concentration was determined using the Lowry method. A total of 50 μg of protein was separated on 4–20% Mini-PROTEAN TGX Stain-Free Protein Gels and electrotransferred under standard conditions (30 min) using Trans-Blot Turbo to a PVDF membrane (all from Bio-Rad, Hercules, USA). The membrane was blocked with blocking buffer containing non-fat milk (Bio-Rad, Hercules, USA). Immunodetection was performed using the goat anti-human DPPIV/CD26 antibody–AF1180 (R&D systems, Minneapolis, USA) 1:1,000—110 kDa and anti-beta actin antibody [AC-15]–ab6276 (Abcam, Cambridge, UK) 1:10,000– 42 kDa. The detection of the target protein was achieved by incubating the membrane with Clarity ECL Western Blotting Substrate and analysed with ChemiDoc XRS system (Bio-Rad, Hercules, USA).

### DPP4 inhibition

We used the FDA-approved DPP4 inhibitor sitagliptin (Sigma Aldrich, St. Louis, USA) to evaluate the effect of DPP4 downregulation on myoblast function. The experiments consisted of two phases: initially, inhibitor toxicity was checked at concentration ranges of 0.1–1,000 µM (data not shown). In the second phase, we evaluated the efficiency of myogenic differentiation of Mb treated with 1 µM, 5 µM or 10 µM sitagliptin maintained throughout the entire procedure. For all experiments, close-to-confluence cell density was used (> 1 × 10^6^/10 cm^2^).

## Supplementary information

Supplementary file1.
