# Supplementary material for: Chromatin and transcriptome changes in human myoblasts show spatio-temporal correlations and demonstrate DPP4 inhibition in differentiated myotubes
Source: Sci Rep. 2020 Aug 31;10:14336. doi: 10.1038/s41598-020-70756-x (PMC7459101; doi:10.1038/s41598-020-70756-x)

Chromatin and transcriptome changes in human myoblasts show spatio-temporal correlations and demonstrate DPP4 inhibition in differentiated myotubes

Tomasz J. Kolanowski[1], Natalia Rozwadowska[1], Agnieszka Zimna[1], Magdalena Nowaczyk[1], Marcin Siatkowski[2], Wojciech Łabędź[3], Ewa Wiland[1], Jacek Gapiński[4], Stefan Jurga[4], Maciej Kurpisz[1]*

1 Institute of Human Genetics Polish Academy of Sciences, Poznan, Poland

2 Institute of Biostatistics and Informatics in Medicine and Ageing Research, Medical Faculty, University of Rostock, Rostock, Germany

3 Department of Spondyloorthopaedics and Biomechanics of Spine, W. Dega University Hospital, University of Medical Sciences, Poznan, Poland

4 NanoBioMedical Center, University of Adam Mickiewicz, Poznan, Poland

Running title: Outcome of chromatin repositioning in myoblasts

*Corresponding author: maciej.kurpisz@igcz.poznan.pl

Supplementary Figure S1. Nuclei morphology in Mb and Mt groups. Nuclei of myotubes after differentiation were significantly smaller (A) and more flattened (B) than undifferentiated counterparts. Example of 3 dimensional reconstruction of the nuclei shape used during analyses(C). Red signals indicate chromosome 2 centromeres, while green signals represent DPP4 loci. Analyses were made using ANNOVA (**** p<0.0001). Bars indicate 5μm.

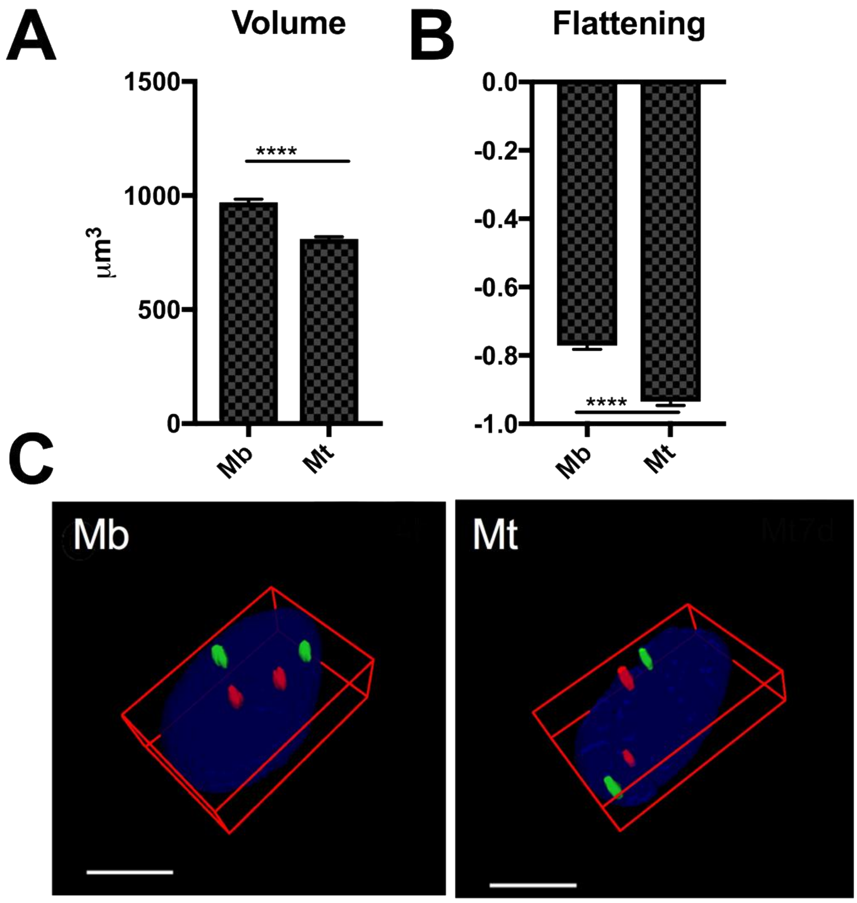

Supplementary Figure S2 Localization changes of selected GOIs before and after differentiation of human myoblasts. Confocal images before and after differentiation for each selected probe (GOI) are shown. GOI probe localization is shown in terms of distribution from the nuclear centre (0.0) to the nuclear periphery (1.0), also called normalized distance index. Loci with increased expression (red) show a general tendency to move towards the middle zones between the nuclear centre and periphery. Loci of genes that did not undergo expression changes (green) resided mainly in the same area. Downregulated genes (blue) had the tendency to reside or move towards extreme zones: periphery (like DPP4) or nuclear centre (in the case of MYH5 and MYH6). Asterisks: * p<0.05; *** p<0.001; arrows show the direction of the observed localization change.

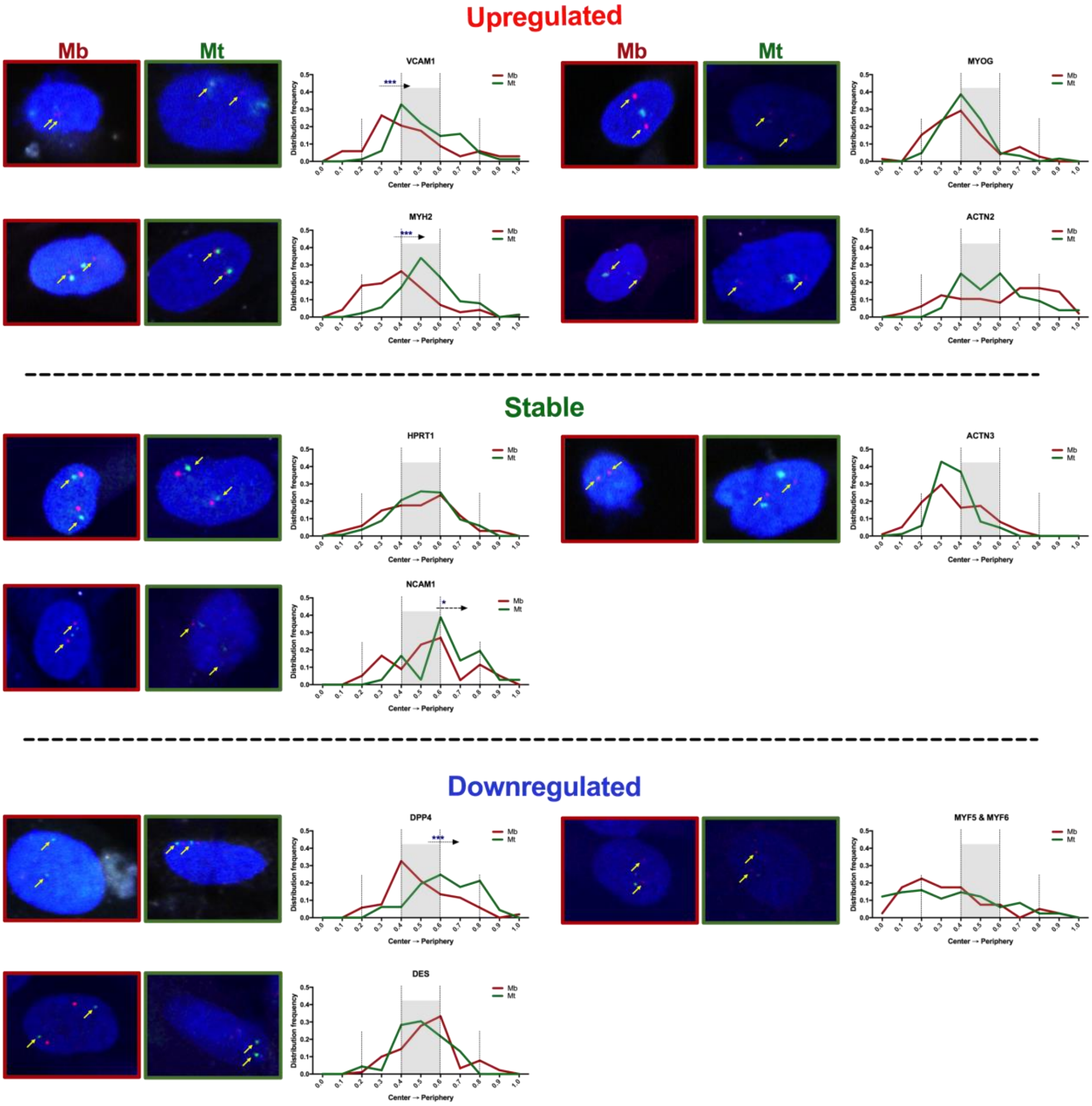

Supplementary Figure S3. High resolution confocal images of the DPP4 (green) positioning in myoblasts(Mb) and myotubes (Mt) using immunoFISH technique. Laminin (A+C) antibody and centromeric probe were stained in red. After 7days of differentiation (Mt group) DPP4 sequence has moved to the nuclear membrane proximity. Physical distance between gene sequence and centromere have decreased as well. Only chosen confocal plane from 3D stack is shown below. Due to this only one signal of DPP4 sequence and centromere can be seen here. Nuclei were counterstained using DAPI (blue). Red bars mark 5 μm.

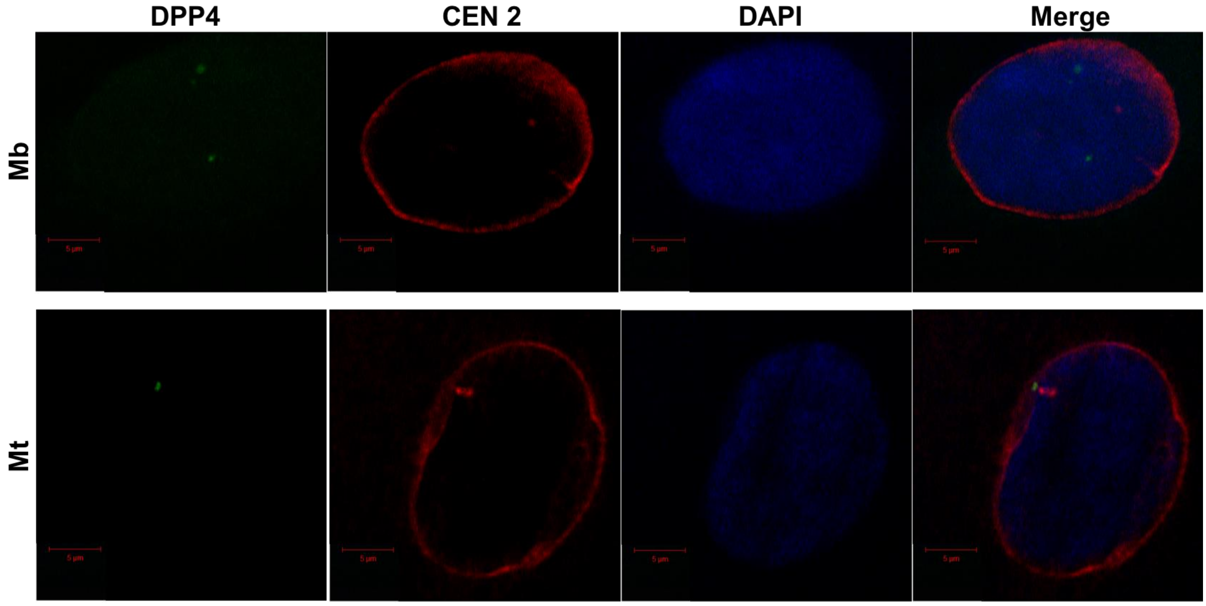

Supplementary Figure S4. A. Charts show changes in localization of studied centromeres in population of myoblasts (Mb, red) and myotubes (Mt, green). We have observed centromeres of chromosomes 1 ($p<0,05$), 2 ($p<0,01$) and 17 ($p<0,001$) to change position from nuclei center towards periphery, preferentially locating in the middle zones. **B.** Scheme of measurements. We have measured distances (yellow arrows) between centromere centers (red points) and nuclear center (yellow cross) in 3D reconstruction of nucleus. These distances were later normalized for each nucleus. Bars indicate 5μm.

A

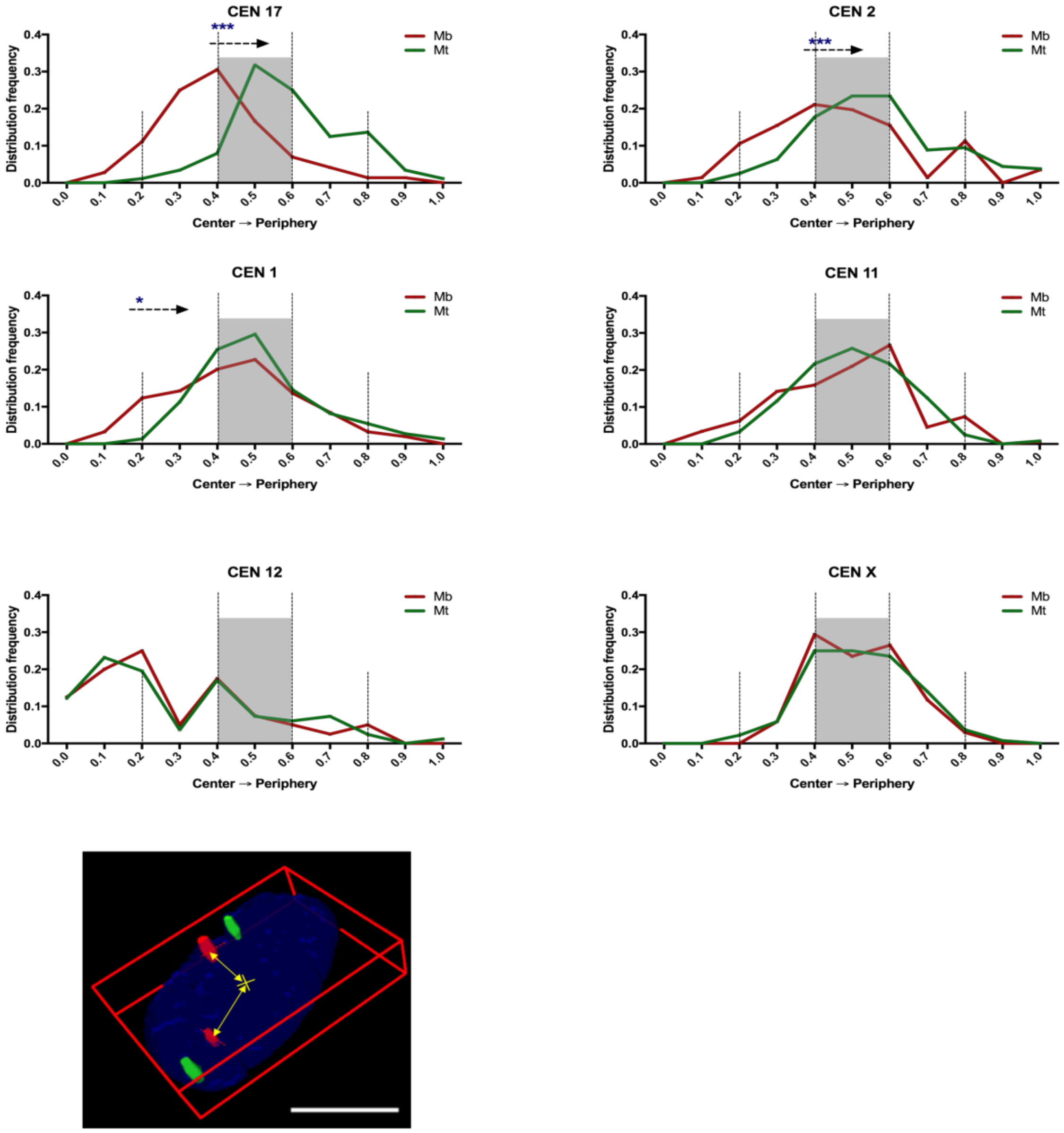

B

Supplementary Table S1. Comparison of distances from the nucleus center for gene-specific probes analyzed in myoblasts (Mb) and differentiated myotubes (Mt)

Probe no.	Gene	Mb		Mt		p Value
		Mean	SD	Mean	SD	
2	NCAM1	0,530	0,179	0,625	0,157	p < 0.05
4	DES	0,529	0,143	0,498	0,122	ns
6	MYOG	0,402	0,160	0,418	0,114	ns
7	ACTN3	0,354	0,146	0,358	0,090	ns
9	MYF5, MYF6	0,326	0,217	0,336	0,235	ns
11	HPRT1	0,488	0,173	0,512	0,143	ns
13	ACTN2	0,596	0,245	0,579	0,176	ns
17	MYH2	0,393	0,177	0,537	0,145	p < 0.001
18	DPP4	0,503	0,166	0,620	0,151	p < 0.001
19	VCAM1	0,443	0,204	0,527	0,149	p < 0.01

NCAM1 - Neural Cell Adhesion Molecule 1, DES -Desmin, MYOG - Myogenin), ACTN3 - alpha-Actinin 3, MYF5 - Myogenic Factor 5, MYF6 - Myogenic Factor 6, HPRT1 - Hypoxanthine Phosphoribosyltransferase 1, ACTN2 - alpha-Actinin 2, MYH2 - Myosin Heavy Chain 2, DPP4 - Dipeptydyl Pepsidase 4, VCAM1 - Vascular Cell Adhesion Molecule 1.

Supplementary Table S2. Distance changes from the nucleus center of centromeric regions (heterochromatin) for selected chromosomes between myoblasts (Mb) and myotubes (Mt)

CEN	Mb		Mt		p Value
	Mean	SD	Mean	SD	
1	0,4462	0,1776	0,5118	0,155	p < 0.05
2	0,4846	0,2008	0,5726	0,1788	p < 0.001
11	0,4842	0,1753	0,5009	0,1392	ns
12	0,2823	0,2212	0,2987	0,2375	ns
17	0,4001	0,1554	0,5895	0,1525	p < 0.001
X	0,5141	0,1247	0,5223	0,1344	ns

Supplementary Table S3. Specification of the probes used in experiments.

Locus-specific probes					
**No.**	**Clone/cat. No.**	**Supplier***	**Gene of interest/ locus**	**Chrom. Localization**	**Working name**
1	RP11-153K4	own	CD56(NCAM1)	11q23.1	BAC2
2	RP11-316O14	own	DES	2q35	BAC4
4	RP11-335O13	own	MYOG	1q31-q41	BAC6
5	RP11-527H7	own	ACTN3	11q13.1	BAC7
6	RP11-14H4	own	MYF5+MYF6	12q21	BAC9
7	RP11-671P4	own	HPRT1	Xq26.1	BAC11
8	RP11-433B10	own	ACTN2	1q42-43	BAC13
9	MYH2-20-RE	Emp. Gen.	MYH2	17p13.1	BAC17
10	DPP4-20-GR	Emp. Gen.	DPP4	2q24.3	BAC18
11	VCAM1-20-RE	Emp. Gen.	VCAM1	1p31-p32	BAC19
Centromeric (satellite) probes					
**No.**	**Clone/cat. No.**	**Supplier***	**Gene of interest/ locus**	**Chrom. Localization**	**Working name**
1	LPE 001R	Cytocell	1	1q12	Chr1
2	LPE 002G	Cytocell	2	2p11.1-q11.1	Chr2
3	LPE 011R	Cytocell	11	11p11.1-q11.1	Chr11
4	LPE 012R	Cytocell	12	12p11.1-q11.1	Chr12
5	LPE 017R	Cytocell	17	17p11.1-q11.1	Chr17
6	LPE 00XG	Cytocell	X	Xp11.1-q11.1	ChrX

* - own - probes produced in our laboratory; Emp. Gen. - probes from Empire Genomics, USA

Supplementary Figure S5. Confirmation of probes specificity. Each probe derived was evaluated for its specificity. Metaphase- FISH experiments were performed as described in Materials and Methods.

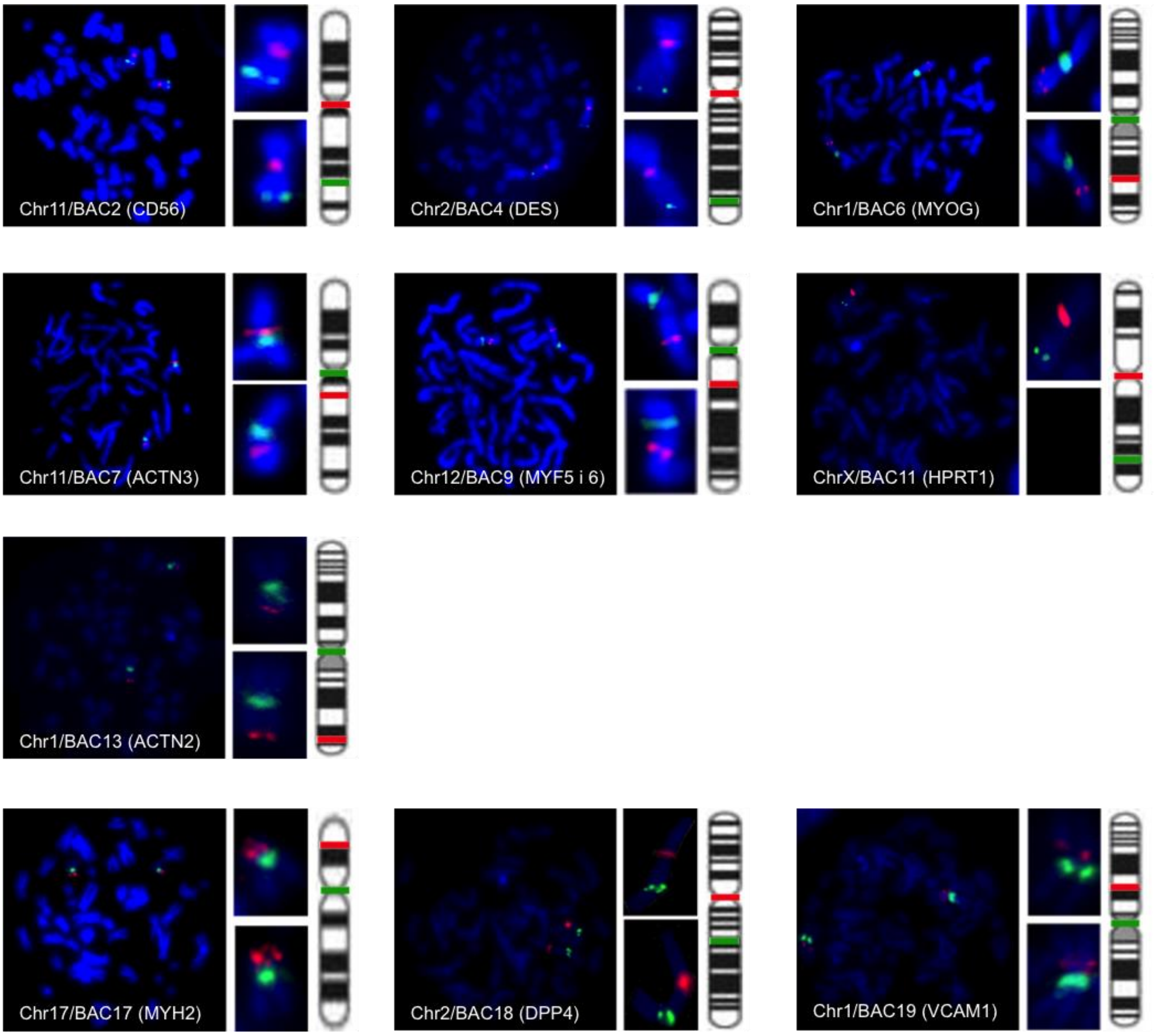

Supplementary Figure S6. Temperature profile of probes denaturation. Experiments with locus-specific probes produced in our laboratory required higher denaturation temperatures and longer time of incubation in comparison to commercial probes. Specific fixation procedure in myoblast samples created thicker samples with preserved 3D architecture. As a result, a higher temperature and longer time was required for specific binding of the probes to DNA. Myo – 3D FISH-prepared slides with myoblast/myotube cells; Metaph – slides with standard lymphocyte culture for probes specificity validation. BAC – experiment set with self-made and commercial centromeric probes; EmpGen – experiments with commercial probes.

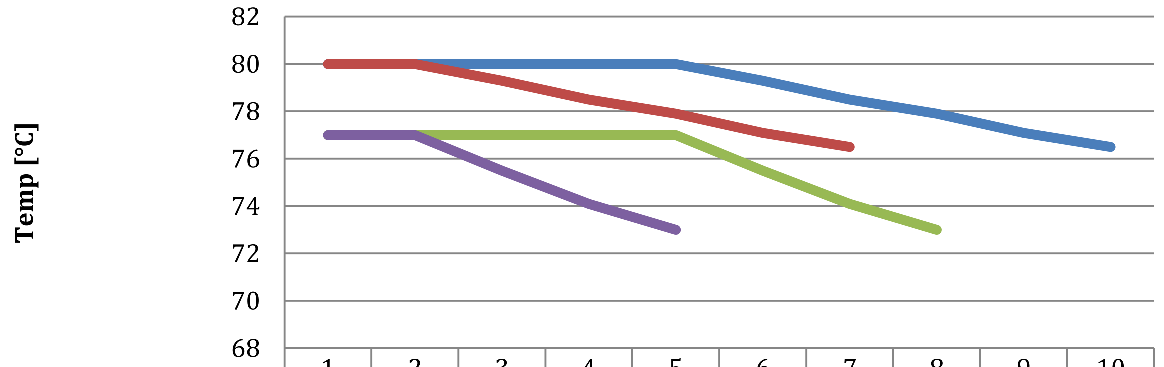

	1	2	3	4	5	6	7	8	9	10
Myo_BAC	80	80	80	80	80	79,3	78,5	77,9	77,1	76,5
Metaph_BAC	80	80	79,3	78,5	77,9	77,1	76,5			
Myo_EmpGen	77	77	77	77	77	75,5	74,1	73		
Metaph_EmpGen	77	77	75,5	74,1	73					

Time [min]

Supplementary Figure S7. Original Full-length blots illustrating resolution of protein product coded by *DPP4* **(A)** and respective reference control of α – ACTN**(B).**

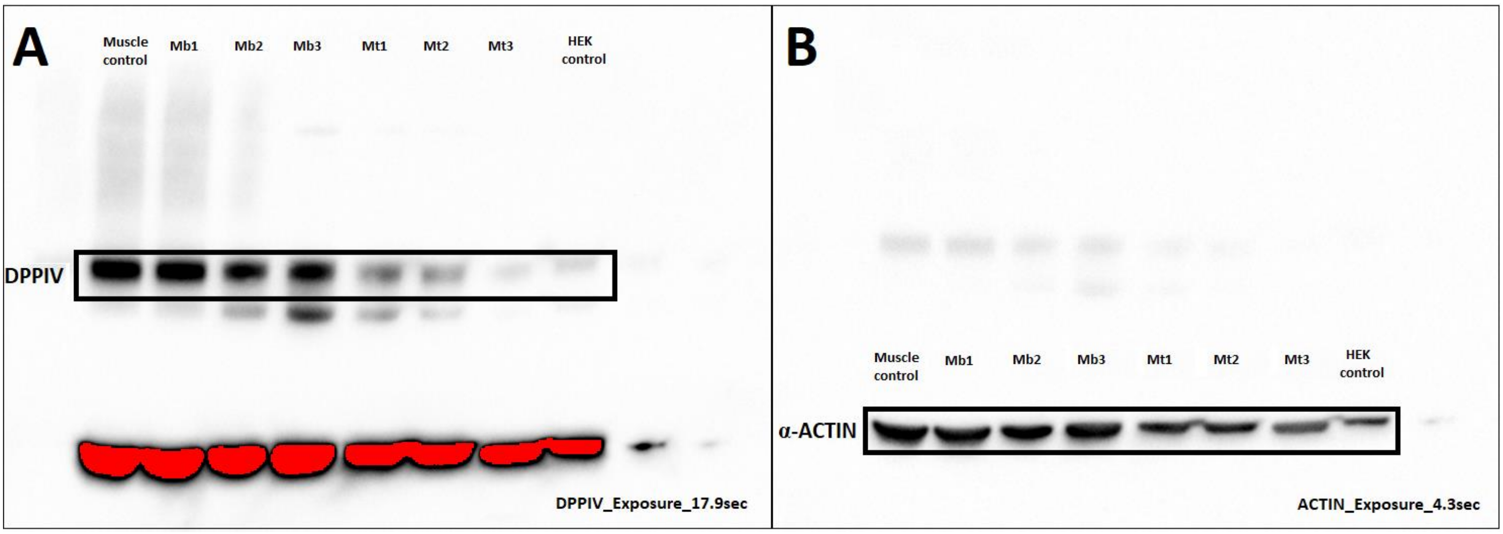

Supplementary Figure S8. Measurements performed for each analyzed nuclei (A) and visualization of selected distances (B). For image clarity not all distances marked in the table have been marked. Nucleus center position was calculated from nuclei signal positioning (DAPI fluorescence). The nucleus border position was defined as a set of points (each with its own coordinates).
While defining the distance, a minimum distance from signal center to the point on a nucleus border was selected. Other distances were calculated from coordinates of signal centers.
Normalization was performed by computationally dividing nuclei volume into 1000 exclusive, isovolumetric and unicentric spheroids, each centered in the core of the nucleus. The measured signal was then computationally distributed to the sphere it belongs to, thus defining its relative distance to the center and nuclei periphery (therefore the range from 0.0 – nuclei center to 1.0 – periphery). The distances between probes (genes or centromeres) were normalized by the nuclei size as well. Flattening was calculated using known ferret distance, nucleus volume and cross-sectional diameter values.

Cen1 & 2 – signal detected from chromosome centromere specific probe; GOI1 & 2 – signal from Gene of Interest loci.

A

**object**	cen1	cen2	GOI1	GOI2	nucleus center	nucleus border
cen1	×					
cen2	✓1	×				
GOI1	✓3	✓	×			
GOI2	✓	✓4	✓2	×		
nucleus center	✓5	✓6	✓7	✓8	×	
nucleus border	✓	✓	✓	✓	✓	×

B

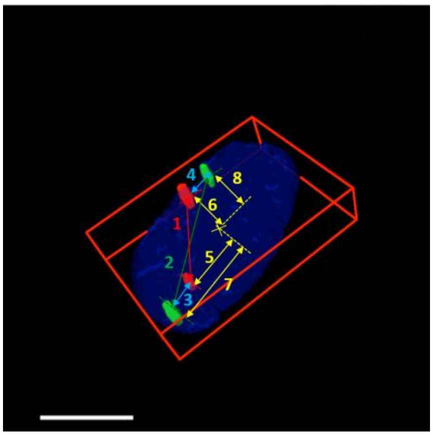

Supplement: Supplementary file 1 — Supplementary file1. [file 41598_2020_70756_MOESM1_ESM.pdf]
